# De novo sequencing and analysis of the transcriptome of two highbush blueberry (*Vaccinium corymbosum* L.) cultivars ‘Bluecrop’ and ‘Legacy’ at harvest and following post-harvest storage

**DOI:** 10.1371/journal.pone.0255139

**Published:** 2021-08-02

**Authors:** María Cárcamo de la Concepción, Daniel James Sargent, Nada Šurbanovski, Richard John Colgan, Marco Moretto

**Affiliations:** 1 Natural Resources Institute, University of Greenwich, Chatham, Kent, United Kingdom; 2 NIAB EMR, East Malling, Kent, United Kingdom; 3 Unit of Computational Biology, Research and Innovation Centre, Fondazione Edmund Mach (FEM), San Michele all’Adige, Italy; University of Naples Federico II, ITALY

## Abstract

Fruit firmness and in particular the individual components of texture and moisture loss, are considered the key quality traits when describing blueberry fruit quality, and whilst these traits are genetically regulated, the mechanisms governing their control are not clearly understood. In this investigation, RNAseq was performed on fruits of two blueberry cultivars with very different storage properties, ‘Bluecrop’ and ‘Legacy’, at harvest, three weeks storage in a non-modified environment at 4 °C and after three weeks storage at 4 °C followed by three days at 21 °C, with the aim of understanding the transcriptional changes that occur during storage in cultivars with very different post-harvest fruit quality. *De novo* assemblies of the transcriptomes of the two cultivars were performed separately and a total of 39,335 and 41,896 unigenes for ‘Bluecrop’ and ‘Legacy’ respectively were resolved. Differential gene expression analyses were grouped into four cluster profiles based on changes in transcript abundance between harvest and 24 days post-harvest. A total of 290 unigenes were up-regulated in ‘Legacy’ only, 685 were up-regulated in ‘Bluecrop’, 252 were up-regulated in both cultivars and 948 were down-regulated in both cultivars between harvest and 24 days post-harvest. Unigenes showing significant differential expression between harvest and following post-harvest cold-storage were grouped into classes of biological processes including stress responses, cell wall metabolism, wax metabolism, calcium metabolism, cellular components, and biological processes. In total 21 differentially expressed unigenes with a putative role in regulating the response to post-harvest cold-storage in the two cultivars were identified from the *de novo* transcriptome assemblies performed. The results presented provide a stable foundation from which to perform further analyses with which to functionally validate the candidate genes identified, and to begin to understand the genetic mechanisms controlling changes in firmness in blueberry fruits post-harvest.

## Introduction

Blueberries belong to the genus *Vaccinium* which includes the cranberries, lilberries and lingonberries. The genus is a member of the Ericaceae family and its species are common and widespread throughout temperate regions of the world [1]. Most blueberry production comes from cultivars derived from *V*. *corymbosum* L. (highbush blueberry; 2*n* = 4*x* = 48), which is native to North America, and are further sub-classified as Southern and Northern high-bush according to their chilling requirement [1]. The fresh consumption of blueberries has significantly increased in recent years due to the release of cultivars with exceptional fruit quality which enhances the eating experience and the perception that blueberries offer nutritional and health benefits [2–4]. Although Europe is the third biggest producer of blueberries globally [5] year-round European consumer demand means blueberries are imported in the off season from Southern-hemisphere production regions including Peru and Chile. As blueberries are a fresh product, the long shipping distances from growing regions to consumer markets mean fruit is in transit for up to six weeks. During transit, fruit softening occurs, significantly reducing fruit quality at consumption [6].

Fruit texture is the most important quality trait from a consumer perspective [7, 8]. Retention of firmness is widely regarded as the main attribute required for long term storage, and as such it ultimately determines the economic success of blueberry varieties in the marketplace [7, 9]. Softening during storage compromises the final eating quality of the fruit, resulting in fewer repeat purchases by consumers and subsequently reduced sustainability for the blueberry industry. Changes in fruit firmness during post-harvest storage are influenced by many different factors including water loss, the effect of temperature and relative humidity, variation in cell turgor, cell wall composition, the effects of high CO_2_ and low O_2_, stem scar size, microstructural changes in cell and tissue layers within the fruit, and skin firmness; all of which have been reported to be genetically regulated [10–14].

Among these factors, water loss, which is primarily influenced by temperature and humidity, has been reported as the main driver of fruit softening [11, 13]. It has been shown that moisture losses below 1.34% in blueberry fruits increased fruit firmness during post-harvest storage [13], whilst significant reduction in firmness was observed in fruit where moisture loss was between 4% and 15% [10, 13]. Other factors shown to have a significant impact on fruit softening include cell organization which impacts texture through changes in cell to cell contact, differences in cell types and size, and thinning or degradation of epidermal layers [11, 15, 16]. Softening during post-harvest cold storage has also been shown to be a consequence of changes in cell wall constituents [17] where a reduction in firmness, or increased softening, was positively correlated with an increase of water soluble pectins and weight loss, and a decrease in cellulose and hemicellulose. Giongo et al., 2013 [7] characterized the texture of a range of blueberry cultivars at different developmental and post-harvest stages and identified a number of different mechanical profiles that described the variation in fruit texture. These profiles included measurements of fruit firmness, and a storage index which was used to describe the potential storage performance of the different cultivars. Positive storage index values characterised cultivars that retained good texture during storage, whilst negative values indicated a loss of texture quality during storage.

Owing in part to the tetraploid nature of its genome, genomics resources for cultivated blueberry have not been developed as quickly as in other fruit species such as peach [18] and grapevine [19]. The genome size of the diploid blueberry has been estimated to be in the region of 600 Mb [20] and a genome sequence consisting of 13,757 contigs assembled from publicly-available sequence data for diploid blueberry has been described [21], but was not made available for public scrutiny. More recently however, the first chromosome-scale genome assembly of the cultivated tetraploid highbush blueberry was released [22] consisting of 48 pseudomolecules containing 1.68 Gb of assembled sequence, and an average of 32,140 protein coding genes per haplotype (128,559 total). The availability of this resource will facilitate further structural and functional genomics studies in cultivated blueberry.

Annotation of the unreleased draft blueberry genome assembly permitted an RNAseq analysis and identification of gene predictions putatively involved in fruit ripening and the biosynthesis of bioactive compounds [21]. Other RNAseq studies in blueberry have characterised changes in gene expression in various tissues under a range of environmental conditions and stresses. These include a study of blueberry root tissue samples with differing tolerance to soil pH changes [23], the identification of genes regulating the anthocyanin content of blueberry fruit during ripening [24], and genes associated with changes in fruit firmness during ripening and harvest [25]. Recently, Zhang et al., 2020 [26] reported the analysis of the transcriptome of the blueberry cultivar ‘Duke’ in response to cold-stress. The authors performed RNAseq experiments on fruit of ‘Duke’ at harvest and following 30 days storage at 0 degrees C, as well as several physiological assessments. Of the 35,060 unigenes recovered, 1,167 genes were upregulated following cold-storage, whilst 685 were down-regulated and a range of genes with Log2FC above +/-2.0 were annotated, including genes associated with plant hormone transduction, carotenoid biosynthesis, glutathione metabolism, starch and sucrose metabolism, protein processing and porphyrin and chlorophyll metabolism. These studies provided insights into changes in blueberry gene expression during cold-stress, but not during industry post-harvest storage protocols. The rationale for the experiments reported here was therefore to begin to understand gene expression changes during conditions likely to be encountered by commercial fruit during post-harvest storage before sale.

To this end, RNAseq was used to determine gene expression profiles in fruits of two blueberry cultivars that exhibit different textural change profiles during post-harvest storage [7]; ‘Legacy’, which retains a firm texture and ‘Bluecrop’, which becomes soft during storage. Samples were profiled at harvest, following cold storage for 21 days and after 21 days cold storage [4°C] followed by 3 days storage at room temperature [21°C]. In order to maximise transcript capture for expression analysis, separate *de novo* unigene assemblies were performed for cvs. ‘Bluecrop’ and ‘Legacy’ and RNAseq reads obtained were mapped to each unigene set independently to identify genes differentially expressed during storage in each of the two cultivars. Comparisons were then performed between the datasets of the two cultivars to identify differentially-expressed genes common between the samples, and those that were unique to the two cultivars. A set of differentially-expressed genes with potential importance to post-harvest storage in blueberry were identified and these are discussed in relation to their roles in the retention of fruit firmness and to previous studies.

## Materials and methods

### Plant material

Three plants each of two Northern highbush blueberry (*V*. *corymbosum*) cvs. ‘Bluecrop’ and ‘Legacy’, were selected for study as they displayed very different textural changes during post-harvest storage [7]. The plants were grown in substrate under tunnels on the Driscoll’s test-plot at East Malling, UK and eight-year-old plants with a recorded crop load of more than six kg per year were used for study. Fruits were carefully harvested by hand by experienced commercial pickers at the same stage of ripeness and development from both cultivars on the same date and following a second stage of careful QC of all material to ensure the same stage of ripeness and development, stored at 4°C in a non-modified environment for 21 days following which they were removed from cold storage and maintained for a further three days at 21°C. Berry tissue was collected immediately following harvest; at 21 days post-harvest, immediately following removal from 4°C cold-storage, and at 24 days post-harvest, following three days at 21°C. Sampled berries were snap frozen in liquid nitrogen and stored at -80°C until use. Three biological replicates were sampled, each of which comprised a random selection of four berries from each sampling point for each cultivar.

### RNA preparation, library construction, and RNA sequencing

Each berry and leaf sample was ground to a fine powder under liquid nitrogen using a mortar and pestle and the powder was collected in 2 ml sampling tubes [LW2420, Alphalaboratories, Hampshire, UK] which were then stored at -80°C until RNA extraction was performed. Total RNA was extracted using the Spectrum^™^ Plant Total RNA kit (STRN250-1KT, Sigma Aldrich, Gillingham, UK) according to protocol A in the manufacturer’s instructions. The concentration and purity of the resultant RNA was measured using a QIAxpert spectrophotometer (9002340, Qiagen, Gilden, Germany). The integrity of the RNA was determined using a qubit 4.0 fluorimeter (Q33226, Thermo Fisher Scientific, Paisley, UK) and samples with RNA integrity number (RIN) values above 6.3 were submitted for RNA-Seq. Library preparation was performed for the 18 fruit samples and two leaf samples using the NEB Next^®^ ultra RNA library prep kit (Biolabs, Inc., Beijing, China) and 150-bp paired-end sequencing was performed by NovoGene Inc (China) using the HiSeq2500 platform (Illumina Inc., San Diego, USA) to yield a minimum of 5.9 GB of data per sample.

### De novo transcriptome assembly and functional annotation

Raw Illumina reads were cleaned and filtered using Trimmomatic version 0.36 [27], trimming sequence with an average quality below 15 in a four bp sliding window. Reads shorter than 36 bp in length following trimming were removed from further analysis. Read QC was performed using Fast QC version 0.11.7 and *de novo* assembly of transcripts was done separately for each cultivar using Bridger version 2014-12-01 [28] with default parameters. CD-HIT [29] was then used with a threshold = 0.99 on ‘Bluecrop’ and ‘Legacy’ transcripts separately to reduce redundancy, and GeneMarkS [30] (GMST) was used to predict protein coding regions of each of the transcript sets using default parameters. Align-back of the original reads to the final unigenes revealed 33% of the raw reads that did not map to the unigene set. These reads were mapped back to the unigenes that were removed from the dataset following the analysis with GMST and unigenes to which more than 50 reads mapped were replaced in the final unigene sets. Protein coding gene annotation completeness was determined using the BUSCO v4.1.4pipeline [31] with default parameters using the gene families set defined for the embryophyta odb10 lineage.

The ‘Bluecrop’ and ‘Legacy’ unigene sets obtained from GMST were used as queries for Blastp [32] against the diploid blueberry transcriptome [https://bitbucket.org/lorainelab/blueberrygenome/src/468455807e4b?at=master], Kiwi transcriptome [http://bioinfo.bti.cornell.edu/cgi-bin/kiwi/download.cgi] and Viridiplantae (Uniref100). Only sequences with an alignment length ≥70% with respect to the database sequence and a sequence similarity ≥70% were retained for further analysis.

### Analysis of unigene expression

Trimmed reads of each sample and treatment were aligned to the ‘Bluecrop’ and ‘Legacy’ *de novo* transcriptome assemblies using Bowtie2 [version 2.3.4] [33] and the read counts for each sample were calculated using featureCounts (1.6.0) [34]. Differential expression analysis was carried out using the Limma empirical Bayes analysis pipeline [35] and voom [36], which estimates the mean variance trend of the log counts to predict the variance and to generate a precision weight to be incorporated in the linear model. Principal components analysis was performed using the Python programming language and the scikit.learn Python package (www.python.org) on Limma-normalized expression values of the genes shared between the two cultivars ‘Bluecrop’ and ‘Legacy’ identified using the reciprocal best blast hit (rbbh) method. Figures were plotted using the matplotlib and seaborn Python packages [37, 38] and VennDiagram package from R Core Team [39]. Unigene annotation was performed using Interproscan, Blastp against the Uniprot Viridiplantae uniref 100 database (using a cut-off of e-value >1*10^-5), and assignment of gene ontology (GO) terms was performed with EggNOG [40]. The distribution of GO functional classifications for the Unigenes was plotted with WEGO 2.0 using default parameters [41].

A double cut off on both p-value and fold change was used to select differentially expressed genes and a p-value < 0.001 and a minimum logFC > 1 were used to classify genes as differentially expressed. Three differential expression analyses were performed; one within each cultivar, and one between the ‘Bluecrop’ and ‘Legacy’ cultivars. The comparison performed between the ‘Bluecrop’ and ‘Legacy’ cultivars was carried out using the results of the rbbh analysis, with the 27,919 genes shared between the two cultivars. The significantly up- and down-regulated genes identified between ‘Bluecrop’ and ‘Legacy’ were grouped into four clusters using matplotlib in Python [37] representing genes up-regulated between harvest and post-harvest for both cultivars, genes down-regulated between harvest and post-harvest for both cultivars, genes up-regulated in ‘Legacy’ and down-regulated in ‘Bluecrop’ between harvest and post-harvest, and finally genes down-regulated in ‘Legacy’ and up-regulated in ‘Bluecrop’ between harvest and post-harvest. The functional annotations assigned to the differentially expressed unigenes were then scrutinised to identify differentially-expressed candidate unigenes with a potential role in regulating textural changes during post-harvest storage. Additionally, the Kyoto Encyclopedia of Genes and Genomes (KEGG) database [https://www.genome.jp/kegg/tool/map_pathway.html] was used to search and reconstruct the pathways of the differentially expressed genes from harvest to post-harvest in ‘Bluecrop’ and in ‘Legacy’. The histogram of KEGG pathways was plotted using Excel.

The normalized expression levels of the candidate unigenes at harvest and post-harvest were used to plot a heat map with the heatmap3 package in R Core Team [39].

### Comparison of unigenes with tetraploid blueberry gene predictions

The ‘Legacy’ and ‘Bluecrop’ assembled unigenes were compared to the nucleotide sequence of the published tetraploid blueberry genome [22] with Blastn (BLAST+ 2.7.1) using default parameters.

### Quantitative real-time PCR validation of RNAseq data

cDNA was synthesised using the Omniscript reverse transcription kit (205111, Qiagen, Hilden, Germany) according to the manufacturer’s recommendations with 500 ng of high-quality RNA used in 20 μl reactions. In total, four candidate genes with significant levels of differential expression in the RNAseq analysis and with relevant functions related to firmness changes during storage for both cultivars were selected for qRT-PCR. These were: unigene_8556 (O-Acytransferase; down regulated in ‘Legacy’ and up regulated in ‘Bluecrop’), unigene_22338 (Cytochome P450; up regulated in ‘Legacy’ and down regulated in ‘Bluecrop’), unigene_20943 (Phloem protein like 2; up regulated in ‘Legacy and in ‘Bluecrop’) and unigene_3134 (Glycoside hydrolase 17; down regulated in ‘Legacy’ and in ‘Bluecrop’). In addition to this, two stably expressed blueberry genes, UBC28 and RH8 [42, 43], were selected for reference gene validation.

The qRT-PCR primers (Table 1) used to study the candidate genes in this investigation were designed using the Primer3 web tool (http://primer3.ut.ee/), checked for primer dimer formation and secondary structures by OligoEvaluator^™^ tool (Sigma-Aldrich, UK) and verified *in silico* using the BLAST function of the Genome Database for *Vaccinium spp* (https://www.vaccinium.org/crop/blueberry) against the ‘Bluecrop’ and ‘Legacy’ unigene sets developed in this investigation and the *Vaccinium* predicted gene set [22]. All qRT-PCR reactions were performed on a Bioer LineGene 9600 qRT-PCR system (Alpha laboratories, Hampshire, UK) using SYBR green as a detector dye. The PCR conditions used were as follows: 95°C for 3 minutes followed by 40 cycles of 30 seconds at 95°C and 1 minute at 60°C. Candidate genes were validated in a total reaction volume of 20 μl containing 4 μl of 1:15 diluted template cDNA, 1x Power up^™^ SYBR^®^ green master mix (A25741, Applied biosystems, Paisley, UK), and 0.4 μM of each forward and reverse primers, whilst for reference genes the reactions contained 0.15 μM of each primer. Relative gene expression was calculated using the 2−ΔΔCt method of Livak and Schmittgen [44]. Expression of each unigene was compared to the geometric mean of the two selected reference genes under harvest and post-harvest storage conditions and normalised against the expression observed in samples collected at harvest. A total of three biological replicates of each cultivar and time point were performed, and the expression levels for each of the samples were calculated based on three technical replicates. Pearson’s correlation was used for the statistical analysis of the data using the R software packages ggpubr and ggplot [39].

**Table 1:** Primer sequences for the four unigenes and two reference genes used for qRT-PCR validation, including primer melting temperatures (T_m_), and reaction efficiencies (E).

Target	Gene name	Name	Sequence (5’ to 3’)	Tm (°C)	Target sequence length (bp)	Reaction efficiency	Reference
Gene of interest	Unigene-8556	Vc8556-F	AAGGAAGTTTAGAGGCCCCG	59.38	70	103%	This study
	(O-acyltransferase)						
		Vc8556-R	TGCTATGGTCGAGTTCTTCAAC	58.41			
Gene of interest	Unigene-22338 (Cytochrome)	Vc22338-F	ATCGGTGTCATCTTCGCAGC	60.81	114	105%	This study
		Vc22338-R	GCCATCTTGTTCTTCGGTGAC	59.54			
Gene of interest	Unigene-20943	Vc20943-F	GACAACTCCTCCACAGAAAAGC	59.45	87	92.2%	This study
	(Phloem protein 2)						
		Vc20943-R	CCCATCAACCCAATACTTCTTCTTC	59.64			
Gene of interest	Unigene-3134	Vc3134-F	TTACAATGTGGGCCTCTCGG	59.97	126	95.6%	This study
	(Glycoside hydrolase17)						
		Vc3134-R	CCCATCTTTGCTACCTTCGAAC	59.32			
Reference gene	RNA helicase-like (RH8)	VcRH8-F	GGTGAATCGAGTAGAACTGCTGGC	63.9	164	102.6%	[42, 43]
		VcRH8-R	AGATTCCTGCATGCACCATTCCGA	69.1			
Reference gene	Ubiquitin-conjugating enzyme (UBC28)	VcUBC28-F	CCATCCACTTCCCTCCAGATTATCCAT	63.8	135	101.8%	[42, 43]
		VcUBC28-R	ACAGATTGAGAGCAGCACCTTGGA	64.2			

## Results

### RNA sequencing and assembly

More than 240 million paired-end reads were generated each for both ‘Bluecrop’ and ‘Legacy’ using Illumina short-read sequencing. After quality control and trimming, approximately 230 million high quality reads remained for each cultivar (detailed in S1 Table). Following *de novo* assembly using Bridger and redundancy reduction with CD-hit, 634,177 ‘Bluecrop’ and 686,520 ‘Legacy’ unigenes were resolved. A total of 233,740 and 249,618 protein coding sequences were identified for ‘Bluecrop’ and ‘Legacy’ respectively from the unigene set using GeneMarkS (GMST) and following Blastp analysis, a final unigene set containing 37,711 ‘Bluecrop’ and 37,093 ‘Legacy’ unigenes was obtained (Table 2). After aligning the original reads back to the final unigene sets, the final number of ‘Bluecrop’ and ‘Legacy’ unigenes was 39,335 and 41,896 respectively. The mean unigene length was 1,081 bp and 1,057 bp with an N50 of 1,419 and 1,392 for ‘Bluecrop’ and ‘Legacy’ respectively. A total of 41.2% of ‘Bluecrop’ and 39.7% of ‘Legacy’ unigenes were greater than 1,000 bp in length (Table 2). A BUSCO analysis of the final unigene sets revealed an 85.3% complete set for ‘Bluecrop’ and an 85.4% complete set for ‘Legacy’, with 4.4% fragmented and 10.3% missing genes and 3.9% fragmented and 10.7% missing genes in the ‘Bluecrop’ and ‘Legacy’ datasets respectively.

**Table 2:** Summary of the transcript data and assembled unigenes for the ’Bluecrop’ and ’Legacy’ datasets.

	Bluecrop	Legacy
Number of transcripts after Bridger	1,055,214	1,106,532
Number of transcripts after CD-HIT	634,177	686,520
Number of transcripts after GMST	233,740	249,618
Number of transcripts after BLAST	34,711	37,093
Number of recovered transcripts	4,624	4,803
Number of Unigenes	39,335	41,896
Number of RBBH Unigenes	27,918	27,918
Total nucleotides of Unigenes	42,539,532	44,300,742
Mean length of Unigenes [bp]	1,081.47	1,057.4
Sequence length > 1000 bp [%]	41.15	39.67
N50 of Unigenes [bp]	1,419	1,392

### Principal component analysis

**Fig 1:**
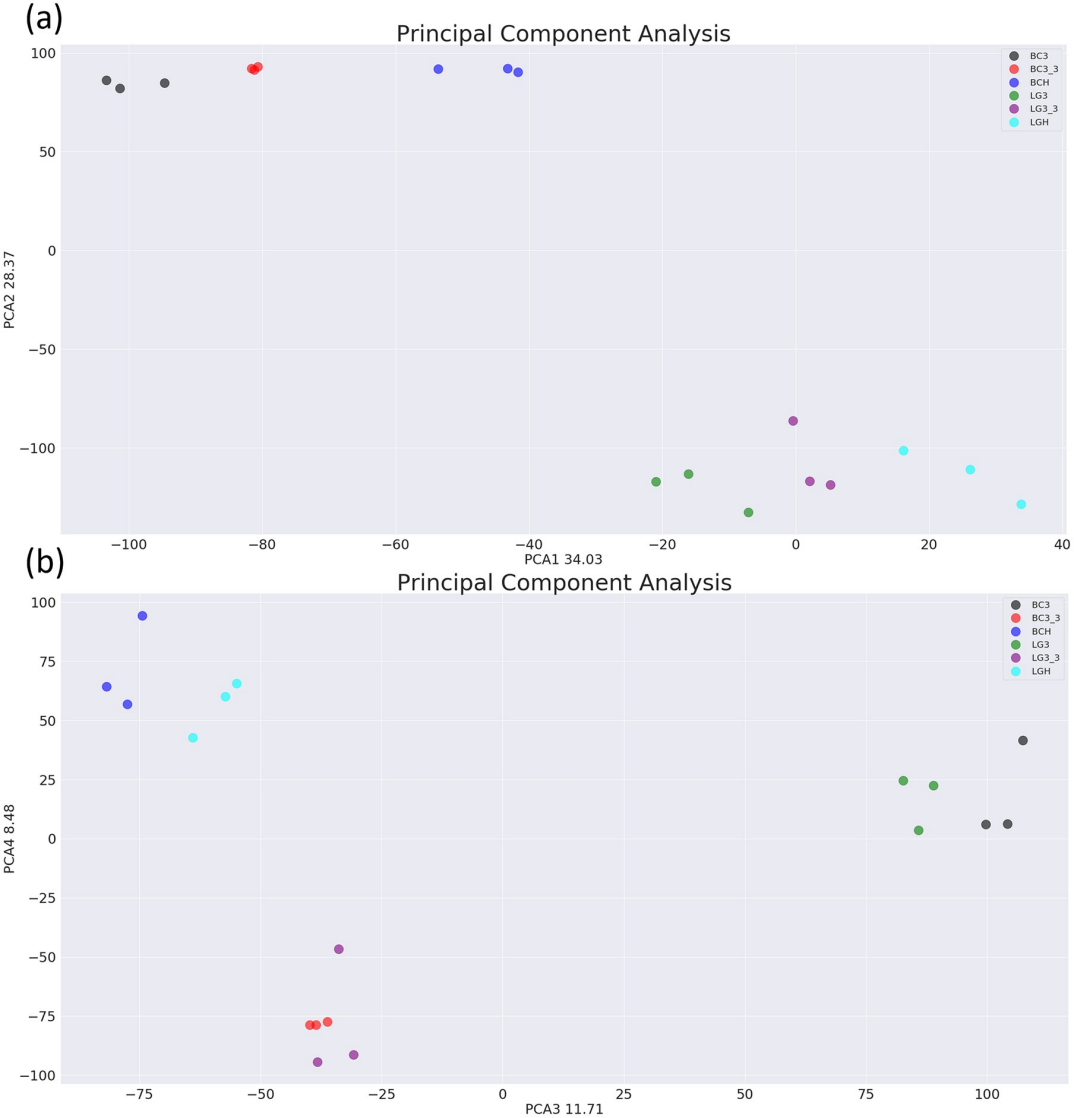
Principal component analysis showing (a) PCA1 and PCA2 (b) PCA3 and PCA4 for ‘Bluecrop’ and ‘Legacy’ fruit at harvest (BCH and LGH), 21 days post-harvest (dph) at 4°C (BC3 and LG3) and 21 dph at 4°C followed by 3 dph at 18°C (24 dph) (BC3_3 and LG3_3).

A principal component analysis was performed which revealed that the maximum variability between samples was explained by tissue (berry vs. leaf) and cultivar differences (Fig 1). Data from ‘harvest’ and ‘24 days post-harvest’ tissues clustered more closely to each other than either did to ‘21 days post-harvest’, indicating that the 4°C temperature at which fruits were sampled after 21 days had a greater effect on gene expression than the post-harvest period itself. As such, the ‘21 days post-harvest’ samples were excluded from further analysis to remove the temperature effect variable from study, and comparisons were made between the ‘harvest’ and ‘24 days post-harvest’ datasets only.

### Sequence annotation

**Fig 2:**
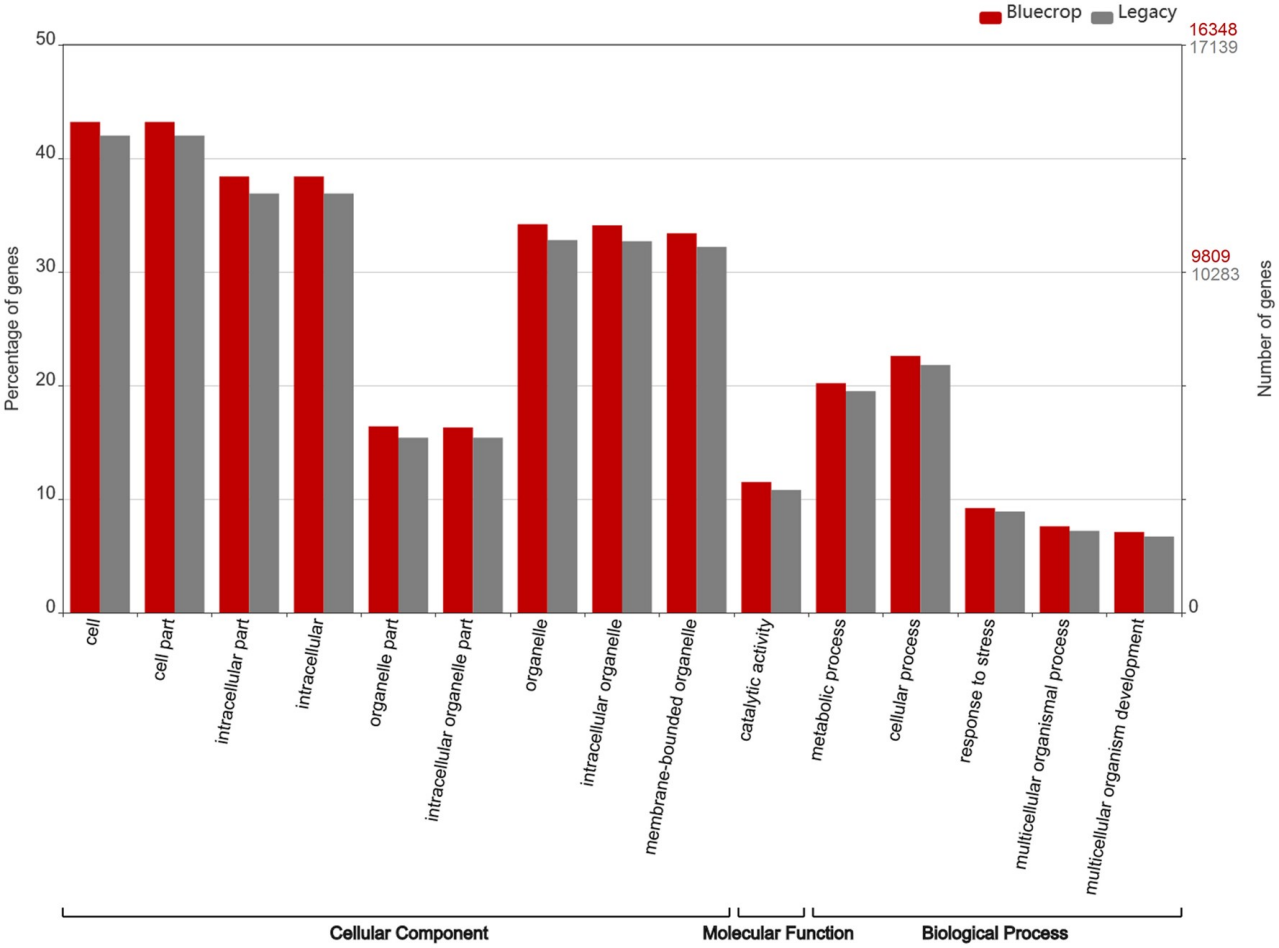
Gene ontology (GO) functional classification of annotated unigenes for ‘Bluecrop’ (red) and ‘Legacy’ (grey) into cellular component, molecular function and biological processes categories. The main y-axis shows the percentage of unigenes annotated whilst the secondary y-axis details the number of unigenes annotated for ‘Bluecrop’ (red) and for ‘Legacy’ (light grey).

A total of 41.4% of the ‘Bluecrop’ and 40.9% of the ‘Legacy’ unigenes were annotated using Interproscan, whilst 45.7% and 45.9% of the ‘Bluecrop’ and ‘Legacy’ unigenes respectively were annotated with Blastp of the Uniprot database. A total of 31,676 and 31,135 gene ontology (GO) terms were associated with all annotated ‘Bluecrop’ and ‘Legacy’ unigenes respectively. The GO classifications revealed a relatively large number of genes associated with ‘cell’, ‘cell part’, ‘intracellular part’, ‘organelle’ and cellular processes for both cultivars (Fig 2). The GO assignments were used to classify the functions of ‘Bluecrop’ and ‘Legacy’ transcripts into three categories, cellular component, molecular function and biological process. A few gene families with extremely high read counts were observed encoding repeat domain proteins associated with stress processes in plants. Pentatricopeptide, LRR (receptor like kinases containing extracellular leucine rich repeat motifs), and WD proteins in particular were all overrepresented.

### Comparison with tetraploid blueberry transcriptome

A total of 38,937 and 36,415 Blastn alignments were returned for the ‘Legacy’ and ‘Bluecrop’ unigene sets when they were queried against the tetraploid *V*. *corymbosum* ‘Draper’ [22] gene prediction set. Alignments were classified by their e-values and their associated alignment length ratios. Almost 80% of the alignments returned an e-value of 0 and an alignment length ratio greater than 0.9 for both cultivars indicating that the ‘Bluecrop’ and ‘Legacy’ unigene sets were representative of the gene complement in the reference sequence. The Blastn alignments for ‘Bluecrop’ and ‘Legacy’ can be found in S2 and S3 Tables respectively.

### Analysis of differentially expressed genes

**Fig 3:**
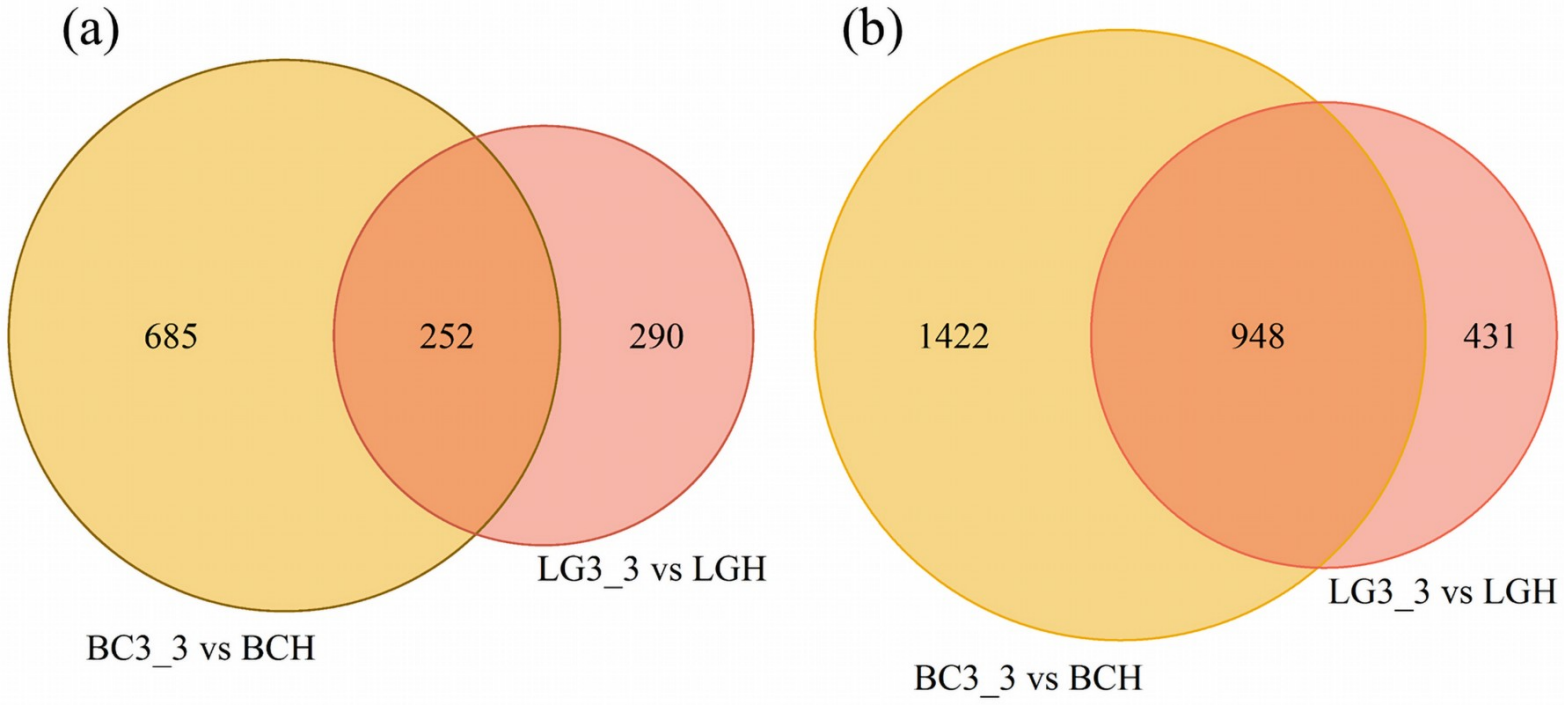
Venn diagram of (a) up-regulated DEGs and (b) down-regulated DEGs for ‘Bluecrop’ and ‘Legacy’ from harvest to 24 dph. BCH and LGH indicates ‘Bluecrop’ and ‘Legacy’ samples at harvest whilst BC3_3 and LG3_3 indicate ‘Bluecrop’ and ‘Legacy’ 24 dph samples.

When gene expression within ‘Bluecrop’ and ‘Legacy’ samples between harvest (H) and 24 days post-harvest (3_3) was compared separately, 937 up-regulated and 2,370 down-regulated genes were identified in ‘Bluecrop’, whilst 542 up-regulated genes and 1,379 down-regulated genes were observed ‘Legacy’. When the differentially expressed gene (DEG) datasets for ‘Bluecrop’ and ‘Legacy’ were compared to each other, 252 genes were identified that were up-regulated between harvest (H) and 24 days post-harvest (3_3) in both ‘Bluecrop’ and ‘Legacy’, whilst 685 genes were up-regulated only in ‘Bluecrop’ and a further 290 were up-regulated only in ‘Legacy’. A total of 948 genes were down-regulated in both ‘Bluecrop’ and ‘Legacy’ between harvest (H) and 24 days post-harvest (3_3), a further 1,422 genes were shown to be down-regulated only in ‘Bluecrop’ and 431 genes were down-regulated only in ‘Legacy’ (Fig 3).

**Fig 4:**
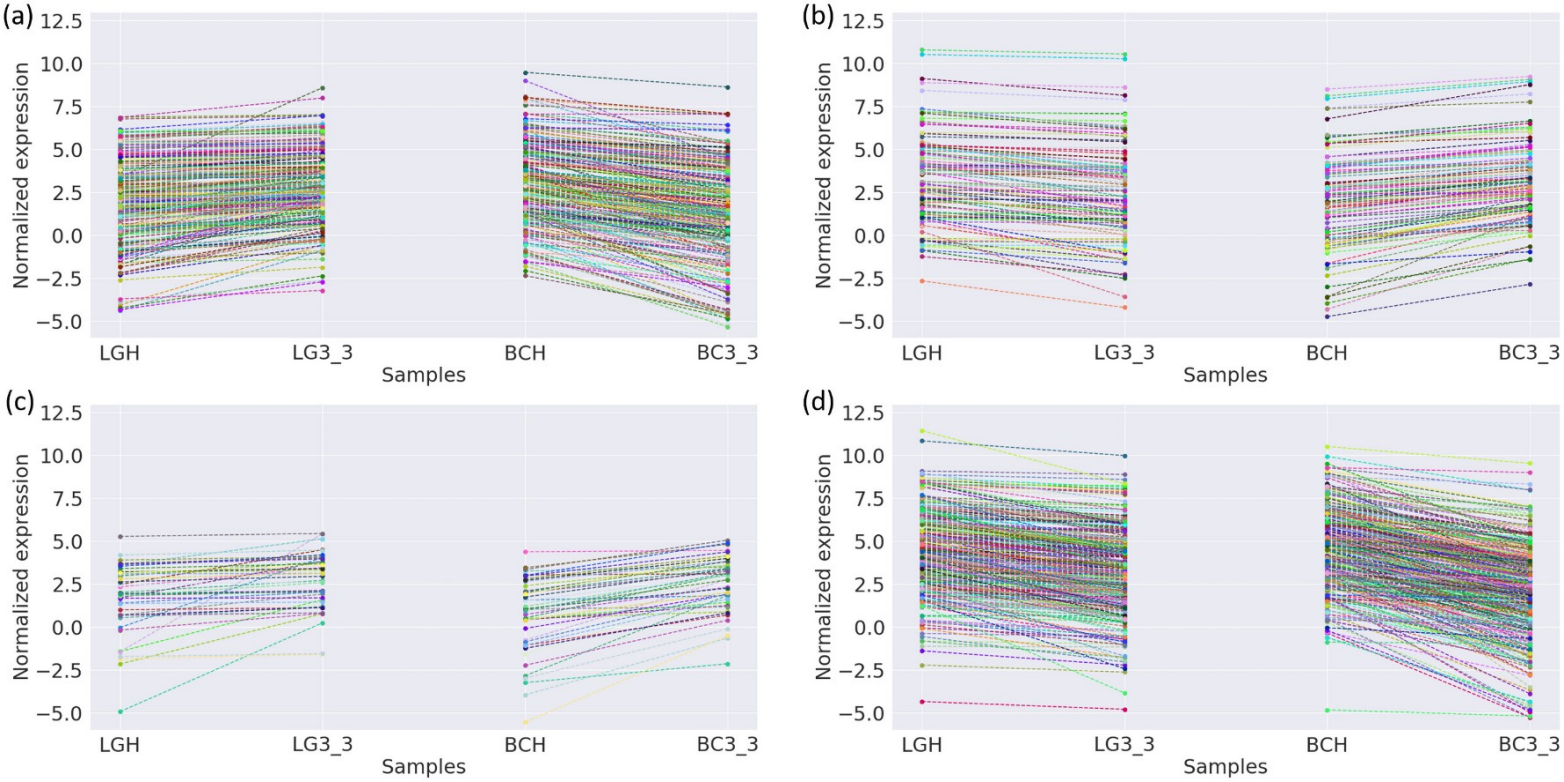
Clustering and classification of genes differentially-expressed within ‘Bluecrop’ and ‘Legacy’ into the four biological expression profiles (a) up-regulated in ‘Legacy’ and down-regulated in ‘Bluecrop’ (b) Down-regulated in ‘Legacy’ and up-regulated in ‘Bluecrop’ (c) Up-regulated in both ‘Legacy’ and ‘Bluecrop’ (d) Down-regulated in both ‘Legacy’ and ‘Bluecrop’. The labels LG and BC indicate ‘Legacy’ and ‘Bluecrop’ whilst H indicates samples from harvest and 3_3 indicates samples from 24 days post harvest.

DEGs were clustered into four model profiles based on the expression levels in ‘Bluecrop’ and ‘Legacy’ when comparing samples at harvest to samples 24 days post-harvest. Between harvest and 24 days post-harvest, a total of 264 DEGs were up-regulated in ‘Legacy’ but down-regulated in ‘Bluecrop’, 103 DEGs were down-regulated in ‘Legacy’ and up-regulated in ‘Bluecrop’, whilst 43 were up-regulated in both cultivars and 355 were down-regulated in both cultivars (Fig 4 and S4 Table).

### Identification of genes associated with physiological changes during post-harvest storage

**Fig 5:**
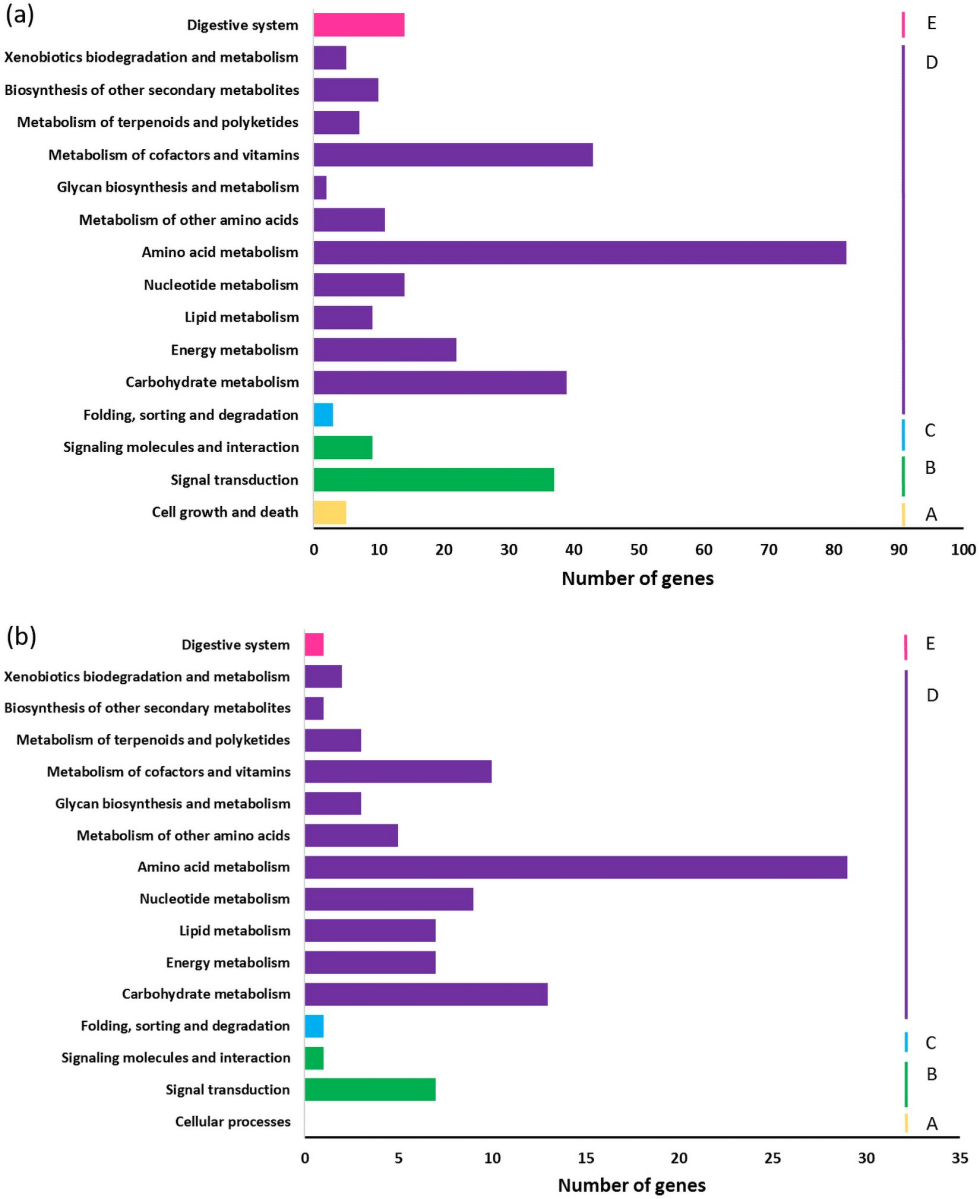
KEGG pathway annotation of DEGs in (a) ‘Bluecrop’ from harvest (BCH) to 24 dph (BC3_3), and (b) ‘Legacy’ from harvest (LGH) to 24 dph (LG3_3). The x-axis shows the number of unigenes annotated and the y-axis details the KEGG pathway category. A) Cellular processes, B) Environmental information processing, C) Genetic information, D) Metabolism and E) Organismal systems.

A total of 170 (5.14%) genes that were differentially expressed between harvest and post-harvest in ‘Bluecrop’ and 59 (3.10%) in ‘Legacy’ could be characterized into corresponding KEGG pathways. DEGs were categorized into 61 pathways for ‘Bluecrop’ and into 50 pathways for ‘Legacy’. The categories with containing larger numbers of DEGs annotated were amino acid metabolism, carbohydrate metabolism and metabolism of cofactors and vitamins for both cultivars. These pathways were followed by energy metabolism in ‘Bluecrop’ and by nucleotide metabolism in Legacy’ (Fig 5).

**Fig 6:**
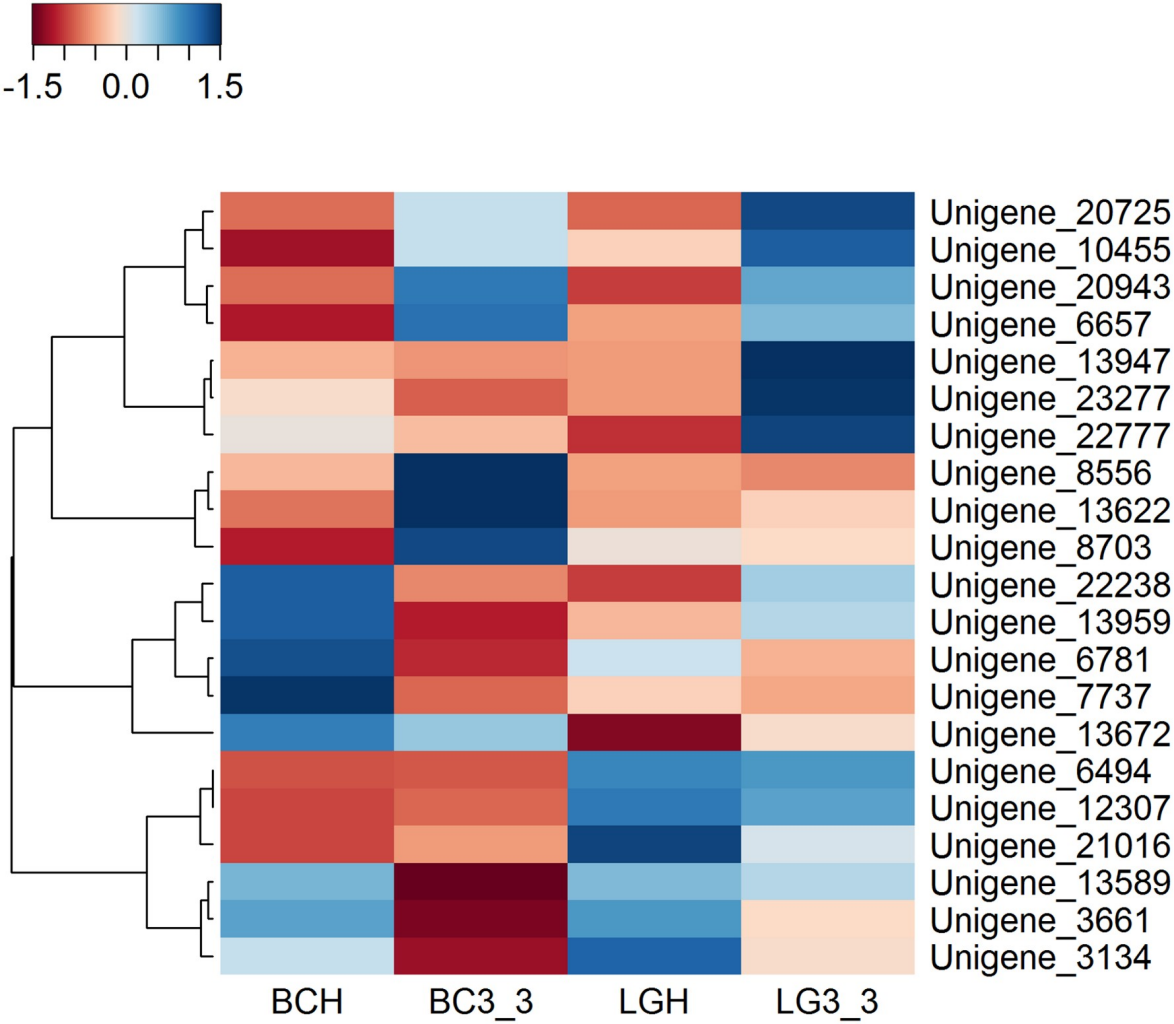
Heat map showing the expression of the 21 candidate unigenes identified as having a potential role in regulating changes in post-harvest firmness in ‘Bluecrop’ and ‘Legacy’. The colour scale represents FPKM normalized expression levels, the rows represent each unigene and the columns represent each cultivar and time point. BC indicates ‘Bluecrop’, LG indicates ‘Legacy’, H indicates harvest and 3_3 indicates 24 days post-harvest.

The unigenes clustered into the following classes; genes associated with biotic and abiotic stress tolerance (Unigene 13622, Unigene 3661); ethylene regulation (Unigene 7737 and Unigene 13947); redox and respiration metabolism (Unigene 10455, Unigene 13622, Unigene 13959 and Unigene 22338); solute transport (Unigene 20943); calcium transport and signalling (Unigene 13589); cell wall metabolism and structure (Unigene 6781, Unigene 3134, Unigene 22777, Unigene 6494, Unigene 21016, Unigene 8703 and Unigene 9136); and synthesis/degradation of wax compounds (Unigene 6657, Unigene 13672, Unigene 8556 and Unigene 12307) (Fig 6).

### Validation of differentially expressed genes by quantitative real-time PCR

**Fig 7:**
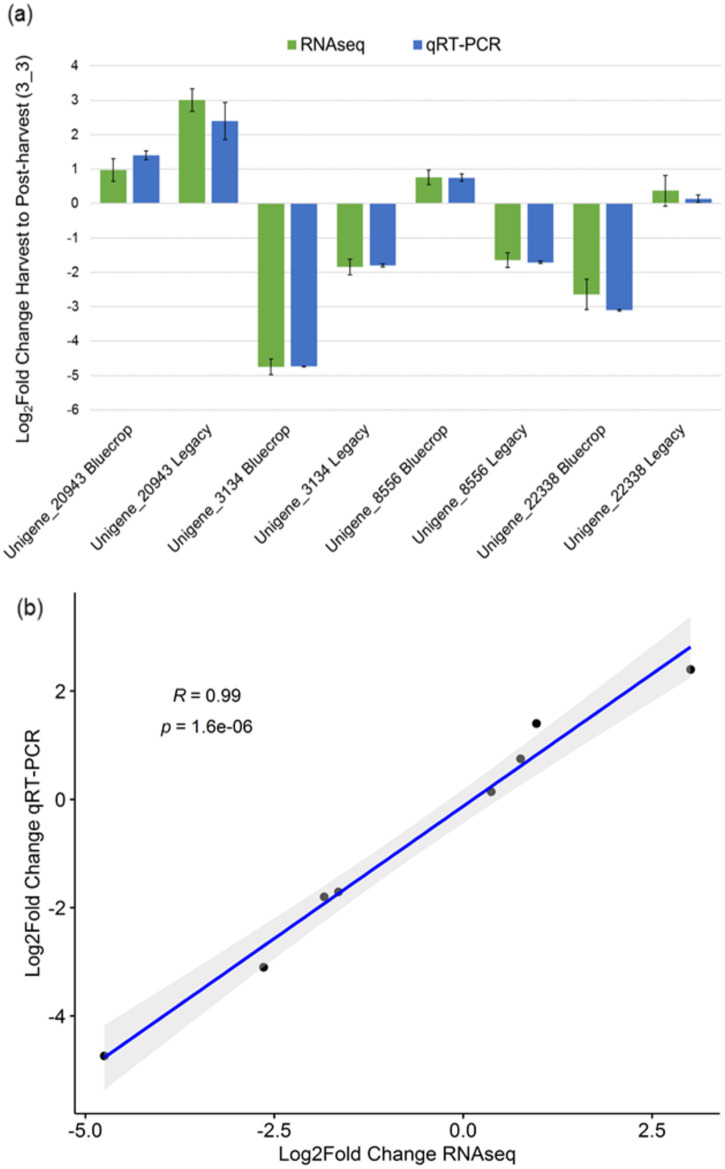
(a) Comparison of the relative expression levels obtained by RNAseq and qRT-PCR for each of the unigenes selected for experimental validation as Log_2_ fold-change from harvest (H) to 24 dph (3_3) for each unigene. The x-axis represents each unigene cultivar combination and the y-axis represents the Log_2_ fold-change from harvest (H) to 24 dph (3_3). (b) Correlation between RNAseq and qRT-PCR (as log_2_ fold change ratio of relative expression at harvest (H) and 24 dph (3_3) in ‘Bluecrop’ and ‘Legacy’) showing the Pearson correlation coefficient and p-values.

In the RNAseq dataset, unigene_8556 was upregulated in ‘Bluecrop’ and down-regulated in ‘Legacy’ between harvest and 24-days post-harvest, unigene_22238 was down-regulated in ‘Bluecrop’ and upregulated in ‘Legacy’ between harvest and 24-days post-harvest, unigene_20943 was upregulated in both ‘Bluecrop’ and ‘Legacy’ between harvest and 24-days post-harvest, and unigene_3134 was down-regulated in both ‘Bluecrop’ and ‘Legacy’ between harvest and 24-days post-harvest (Table 3). Patterns of gene expression following analysis by qRT-PCR were in general agreement with the results from the RNAseq analysis and a linear regression analysis calculating Pearson’s correlation factor revealed a strong correlation (R^2^ = 0.99; p ≤ 0.001) between the log fold-change values observed in the RNAseq data and those observed in the qRT-PCR data (Fig 7).

**Table 3:** Detail of the 21 differentially-expressed unigenes identified as having a potential role in regulating changes in post-harvest firmness.

Unigene ID	LogFC	Pvalue	Interpro ID	Description	E Value
Down regulated in BC and LG					
Unigene_7737	-5.53	7.80E-06	IPR001471	AP2/ERF	3.15E-26
Unigene_3661	-4.87	5.60E-06	IPR003657	WRKY domain superfamily	4.10E-24
Unigene_13959	-3.77	6.92E-08	IPR001117	Multicopper oxidase-cupredoxin	4.40E-23
Unigene_13589	-2.95	1.50E-05	IPR006769	Calcium uniporter	3.90E-52
Unigene_6781	-2.93	3.68E-05	IPR000757	Xyloglucan endo-transglycosylse	4.80E-63
Unigene_3134	-2.91	4.31E-07	IPR000490	Glycoside hydrolase fam 17	1.50E-13
Up regulated in BC and LG					
Unigene_13622	4.79	0.0008	IPR001878	Zinc finger family	7.20E-04
Unigene_6657	1.285	6.22E—05	IPR001330	Terpenoid cyclase	2.90E-08
Unigene_10455	1.284	0.00085	IPR001128	Cytochrome P450	1.40E-12
Unigene_20943	-2.05	0.00057	IPR025886	Phloem protein 2-like	4.70E-37
Unigene_20725	-1.98	8.16E-06	IPR006045	Cupin 1	1.40E-34
Up regulated in LG					
Unigene_23277	-7.69	1.10E-05	IPR014044	CAP domain	6.40E-15
Unigene_13947	-7.18	5.11E-06	IPR001471	AP2/ERF	1.40E-11
Unigene_22777	-6.09	0.00013	IPR006706	Extensin	2.30E-06
Unigene_13672	-5.31	2.12E-05	IPR001906	Terpene synthase	1.40E-28
Unigene_22238	-3.01	0.0003	IPR001128	Cytochrome P450	1.20E-08
Up regulated in BC					
Unigene_12307	4.41	7.33E-06	IPR001906	Terpene synthase	8.90E-45
Unigene_6494	3.85	0.00074	IPR004993	GH3 family	1.50E-93
Unigene_21016	2.693	0.00014	IPR007118	Expansin	5.50E-31
Unigene_8703	2.54	2.77E-06	IPR000743	Glycoside hydrolase fam 28	1.30E-63
Unigene_8556	2.42	1.44E-06	IPR009721	O-acytransferase WSD1	3.40E-22

## Discussion

Here we present analyses of gene expression changes that occurred in the fruit of two blueberry cultivars, ‘Bluecrop’ (known for poor firmness retention) and ‘Legacy’ (known for good firmness retention) during post-harvest storage. Whilst a genome reference sequence for tetraploid blueberry cultivar ‘Draper’ recently became available [22], which provides a reliable genomic resource for transcriptomic analysis, separate *de novo* transcriptome assemblies were performed here for ‘Bluecrop’ and ‘Legacy’ to avoid multiple- and/or mis-alignments within unigenes produced from a hybrid or reference assembly. Overall, the number of transcripts the ‘Legacy’ and ‘Bluecrop’ unigene sets resolved was 41,896 and 39,335 respectively, comparable to those of the published genome that contained an average of 32,140 gene predictions per haplotypes, and the majority of the assembled transcripts returned highly similar, or identical matches to predicted genes within the ‘Draper’ reference genome [22].

The data presented here revealed that cell, cell part, intracellular, membrane-bound organelle part, cellular and metabolic processes, and catalytic activity were the most represented GO categories within the assembled unigene set (Fig 2). These categories were strongly represented in other studies of ‘Bluecrop’ [45, 46] as well as in studies of the cultivars ‘Northland’ [24], ‘O’Neal’ [21] and ‘Duke’ [26]. The vast majority of factors affecting post-harvest quality are under strong genetic control [47] and ‘Bluecrop’ and ‘Legacy’ previously displayed significant differences in their response to prolonged periods of low-temperature post-harvest storage [7], resulting in very different post-storage texture profiles of the two cultivars. A recent study of Zhang et al., 2020 [26] used the cultivar ‘Duke’ which previously displayed texture characteristics very similar to ‘Legacy’ [7] at harvest, but which displayed a significant decline in texture during post-harvest storage. The analysis of differentially expressed genes in ‘Duke’ identified 1,167 upregulated, and 685 down-regulated genes following 30 days storage at 0°C, which contrasted to the 2,370 down-regulated and 937 up-regulated, and 1,379 down-regulated and 542 up-regulated genes in ‘Bluecrop’ and ‘Legacy’ respectively following 21 days storage at 4°C and three days at 18°C in this study.

Validation by RT-qPCR has been used widely in transcriptomic studies of horticultural species to confirm the reliability of RNAseq data [26, 48, 49]. In this investigation, primer pairs were designed for four candidate genes of interest and successfully validated the RNAseq results obtained, revealing a highly significant correlation between gene expression profiles obtained by RNAseq and RT-qPCR analysis, and giving confidence in the RNASeq dataset as a whole. Here, a greater number of down and up regulated genes was observed in ‘Bluecrop’ than ‘Legacy’ [Fig 3] in the analysis of the RNASeq data, suggesting ‘Bluecrop’ fruit is less well adapted to the physiological stresses associated with prolonged storage at 4°C and subsequent shelf life [21°C] than ‘Legacy’, as reflected in a greater decline in fruit quality and texture attributes observed in ‘Bluecrop’ over ‘Legacy’ [7]. Numerous DEGs associated within diverse cell, molecular, and biological pathways were identified as candidate unigenes with a potential role in firmness changes during storage in this study. Most of the DEGs observed between harvest and post-harvest in ‘Bluecrop’ and in ‘Legacy’ were predominantly categorized into amino acid metabolism, carbohydrate metabolism and cofactor and vitamins metabolism, followed by energy metabolism in ‘Bluecrop’ and by nucleotide metabolism in ‘Legacy’ [Fig 5]. Furthermore, most of the DEGs identified between harvest and post-harvest between both cultivars were putatively involved in cell wall metabolism, composition of the skin wax layer, adaptation to abiotic stress and solute transport. Zhang et al., 2020 [26] found membrane lipid metabolism, proline, glutathione and flavonoid metabolism, and hormone biosynthesis and signal transduction as the main pathways in which genes were differentially expressed in ‘Duke’, during storage at 0°C. Whilst many of the categories of DEGs observed were similar between the two studies, the differences observed in gene expression profiles between those reported here for ‘Bluecrop’ and ‘Legacy’, and those reported for ‘Duke’ most probably reflect the differences in the temperature storage protocols employed and most importantly differences in the point of sampling between the two studies. The post-harvest sampling conditions of [26] more closely resembled the sampling point in this investigation immediately following post-harvest storage when fruit was still at 4°C. Zhang et al., 2020 [26], used a temperature of 0°C, and whilst that has also been used in other blueberry studies to compare the fruit quality including firmness at different temperatures and days post-harvest [10, 11], it is not representative of temperatures in retail or home refrigeration settings.

Scrutiny of the differentially expressed transcripts in ‘Bluecrop’ and ‘Legacy’ was focussed on genes that could have played a putative role in the stress response during post-harvest cold-storage, and genes that could play a role in the retention of texture and fruit quality. Previous studies have shown that genes involved in determining the composition of the wax layer [bloom] in blueberry, as well as those involved in cell wall metabolism, adaptation to biotic or abiotic stress, calcium and solute transport could all play a role in maintaining good fruit quality during post-harvest storage, and from these classes, a set of 21 differentially-expressed candidate genes were characterised [Table 3].

### Epicuticular wax metabolism

The waxy layer representing the bloom of blueberries is a key protective mechanism against a range of abiotic stresses, including moisture loss and temperature fluctuations, and has been shown to reduce deterioration in fruit quality during post-harvest storage [50]. Variations in the composition of the wax compounds in plant cuticles can affect their mechanical properties under storage [51–53] and these variations have been correlated with textural changes in blueberry [54], pepper and tomato [55–57] fruits during storage. Unigene 8556 shown to have high homology to wax ester synthase/diacylglycerol acyltransferase [WSD1], an enzyme which catalyses the synthesis of wax ester compounds in the stem, flowers and leaves of *Arabidopsis* [58]. Acyltransferase genes have been reported to be strongly upregulated in *Arabidopsis* in response to abiotic/biotic stress [59] and in *Euruca vesicaria* seedlings in response to drought stress [60]. ‘Bluecrop’ softens more rapidly during storage than ‘Legacy’ [7], indicating a greater rate of moisture loss in ‘Bluecrop’ fruits during storage [13]. Here, unigene 8556 was highly down-regulated in ‘Legacy’, but up-regulated in ‘Bluecrop’, suggesting fruit of ‘Bluecrop’ may be triggering stress response pathways and increasing wax production in response to moisture loss during extended exposure to low temperatures and reduced % relative humidity experienced in post-harvest storage.

Cytochrome P450 enzymes are a large family of haem-containing monooxygenases that catalyse an extensive range of chemical reactions including the biosynthesis of plant hormones and defensive compounds. Cytochrome P450 enzymes complex oxygen molecules to a heam group, and the metal-oxygen complex can oxidise their substrates using NADH or NADPH as co-factors [61]. Zhang et al. [26] reported the up regulation of cytochrome-P450 oxidases in blueberries induced by cold-temperatures including *cytochrome-P450-90A1-*(*CYP90A1*) and *cytochrome-P450-85A2-*(*CYP85A2*) responsible for facilitating secondary metabolite biosynthesis and Brassinosteroids biosynthesis; while *cytochrome P450 707A* (*CYP707A*), encodes a cytochrome P450 involved in terpenoids and polyketides synthesis that form part of the ABA metabolic pathway. Monoterpene oxidases are induced by the abiotic stresses that blueberries are exposed to during harvesting and storage, and Unigene_22238 represents class E cytochrome P450 proteins that includes cytochrome P450 CYP71 that functions as a monoterpene oxidase [62] acting on terpenoid substrates. Hamberger and Bak [63] identified 14 terpenoids associated with blueberry flavour including linalool, limonene, 4-terpineol, nerol, geraniol that were most abundant in green-stage underripe fruit. Monoterpene oxidases CYP71 act on terpenoids in a step wise oxidation manner to form a multitude of triterpenes [64]. Triterpenes are an important fraction of the cuticle wax in blueberry fruits, and have been described as key compounds associated with moisture loss and softening rates during storage [54]. Unigene 22238 was upregulated in ‘Legacy’ but not in ‘Bluecrop’, suggesting that it may contribute to an increased production of secondary metabolites in ‘Legacy’ fruits during post-harvest storage, in particular those related to cuticular wax triterpenes associated with preservation of skin integrity and a reduction of moisture loss, that may impact on fruit texture during post-harvest storage.

### Cell wall metabolism under post-harvest storage

Changes in cell wall structure during development and ripening occur due to degradation of cell wall constituents primarily through the disassembly of the cellulose-hemicellulose network [7, 65–67]. The cellulose and hemicellulose matrix comprise microfibrils linked by hydrogen bonds, that increase the strength of the cell wall. In addition, a pectin matrix interlaces the cellulose-hemicellulose backbone conferring cell wall adhesion [25]. Thus, cell wall modifications and disassembly form part of the regulation of fruit softening, which is a process that has been well characterised in blueberries [17, 68, 69], apple [70], peach [71], strawberries [72, 73] and tomatoes [74, 75].

Glycoside hydrolases have been reported to affect blueberry firmness during ripening and post-harvest stages [25, 26] and their enzymatic activity depends on their specificity to individual carbohydrate components forming the cellulose (homopolymer of glucose) and hemicellulose (xylans, glucans, xyloglucans, callose, mannans and glucomannans) structures [14, 76, 77]. In this investigation, homologues of three glycoside hydrolases; GH17 (Unigene 3134), GH3 (Unigene 6494) and GH28 (Unigene 8703) were shown to be differentially-expressed during post-harvest cold-storage. The GH17 family encodes endoglucanases which degrade cellulose structures [77] and play a role in cell wall degradation in blueberry fruit and banana during ripening stages [25, 78]. Down-regulation of unigene 3134 [GH17] in ‘Bluecrop’ and ‘Legacy’ suggests a reduction in activity of this glycoside hydrolase during cold storage, supporting the findings of [25, 66] who reported reduced glycoside hydrolase activity in blueberry fruits during cold storage. Reduction in activity may be purely temperature related or possibly an adaptation mechanism triggered in both cultivars in response to post-harvest cold-storage [25, 66]. GH3 is a xylosidase enzyme [77] with a role in degrading cell walls and contributing to softening in blueberry fruits [14, 68], whilst GH28 showed high homology to a polygalacturonase enzyme which degrades pectin by hydrolysing the homogalacturonan backbone of the cell wall [79]. This family of enzymes has been shown to be upregulated during post-harvest storage and to a play a role in the softening of blueberry fruits and cell wall degradation [69]. Both GH3 (Unigene 6494) and GH28 (unigene 8703) were highly upregulated in ‘Bluecrop’ and down regulated in ‘Legacy’ during cold storage and thus may play a role in faster cell wall degradation in ‘Bluecrop’, leading to a greater degree of softening.

The role of expansin proteins in cell wall loosening has been well characterised in *Arabidopsis* [80], tomato [81, 82], strawberry [83–85], peach [86] and kiwi fruits [87] where its activity is upregulated during the softening of ripening fruits [88]. Expansins initiate cell wall loosening and extension through the breakage of hydrogen bonds between cellulose and hemicellulose molecules, in particular xyloglucans [89, 90], and their expression is regulated by cross-talk between many plant growth regulators including abscisic acid, indol-3-acetic acid, auxins, brassinosteroids, cytokines, ethylene [88]. Expansin activity has been shown to be enhanced by low pH, and low temperatures in an absence of an ethylene peak during storage [87]. Unigene 21016 was shown to be highly upregulated in ‘Bluecrop’ and down regulated in ‘Legacy’ during post-harvest storage, suggesting that expansins expression in ‘Bluecrop’ may contribute to the increased fruit softening observed by Giongo et a., 2013 [7], as has been reported in kiwi [87] and in tomato [82], where overexpression of expansins produced softer fruit and silencing was correlated to an increase of firmness and extended shelf life.

### Abiotic stress and solute transport

Adaptation to abiotic stress in plants include the control of cell turgor, the induction of cell signalling pathways, an increase in respiration rates and deployment of protective mechanisms against Reactive Oxygen Species (ROS). The APETALA2/ETHYLENE RESPONSE FACTOR (AP2/ERF) is one of the mediators to plant external abiotic responses and developmental processes [91–94], and has been reported to regulate cold adaptation responses in the flower buds of ‘Bluecrop’ [95], and in cold-stored blueberry fruit of ‘Duke’ [26]. Unigene 7737 and Unigene 13947 display high homology to AP2/ERF and were found in differentially regulated clusters in this investigation, suggesting that members of this family of transcription factors may be repressed or induced under the same environmental conditions. AP2/ERF homologs in potato (CIP353) conferred low-temperature acclimation to tubers exposed to long-term cold-storage [93], whilst in tomato the overexpression of PTi4 encoding an AP2/ERF induced a family of expansin genes linked to roles in cell wall integrity [96]. AP2/ERF genes have also been reported to regulate the adaptation to osmotic, water stress and drought tolerance in *Arabidopsis* [97], and osmotic differences between protoplast and the apoplast have been shown to drive changes in turgor pressure in plant cells [98], changes which are linked to post-harvest softening in apple [99] and softening in blueberries [10, 12, 15]. ‘Legacy’ exhibits a greater retention of firmness than ‘Bluecrop’ during post-harvest cold-storage [7] and unigene 13947 was upregulated in ‘Legacy’, and down-regulated in ‘Bluecrop’ suggesting that its expression may play a role in the maintenance of firmness in blueberry fruits during post-harvest cold-storage.

The mitochondria play an important function in buffering cytoplasmic calcium, with mitochondrial calcium uniporters (MCU) acting as calcium sensors and active channel protein for calcium uptake [100–102]. Mitochondrial uptake plays a role in ATP synthesis and regulating calcium concentration in the cytoplasm [Ca]_cyt_ which is essential for regulating its role as a secondary messenger in response to abiotic stress. Moreover, [Ca]_cyt_ plays a role in regulating fruit quality, specifically by increasing the resilience of fruit to low temperature stress by enhancing the energy status of the cells and maintaining osmotic conditions [103]. Unigene 13589 displayed homology to mitochondrial calcium uniporters [MCU] and was down regulated during post-harvest storage for both cultivars, which may cause loss of mitochondrial function and reduced cellular calcium uptake during post-harvest cold-storage, contributing to fruit quality deterioration during storage.

### Phloem lectin proteins

Lectins are non-enzymatic proteins that reversibly bind carbohydrates. Unigene_20943 encodes a putative Phloem Protein 2 (PP2), one of the most abundant protein lectins translocated in the phloem. PP2 can exert effects over long distances; it is synthesised in companion cells before being transported to sieve cells where it is involved in structure and maintenance [104]. Confocal scanning laser microscopy coupled with subcellular fluorescent markers have located PP2 to mitochondrial membranes where it is postulated that it is embedded within the sieve element clamps that facilitate the fixing of organelles to each another [105]. Such PP2 proteins have been reported to immobilize microorganisms and fungi to the cross-linked filaments sealing of the wounded sieve tube, thus facilitating wound sealing and anti-pathogenic responses [106]. Unigene 20943 was upregulated in both ‘Bluecrop’ and ‘Legacy’ during post-harvest storage suggesting that its expression may play a role in the maintenance of the structure of cellular organelles, promoting wound healing and preventing pathogen colonization. Such actions could therefore play a role in maintaining blueberry fruit quality during storage.

## Conclusions

Transcriptional changes during post-harvest storage in ‘Bluecrop’ and in ‘Legacy’ was observed in this study to occur in genes with roles in catalytic activity primarily located in the cell, intracellular parts and membrane-bounded organelles. Additionally, differential expression was observed in genes involved in cell wall metabolism, synthesis of wax compounds and biotic and abiotic stress between harvest and post-harvest in the two cultivars studied. In this initial study, a set of differentially regulated candidate genes were identified between ‘Bluecrop’ and ‘Legacy’ that may have a role in the observed differences in post-harvest fruit quality reported between these two cultivars. The data presented here provides a strong foundation for future focussed studies of the role of these genes in relation to specific physiological changes that occur during post-harvest cold-storage in blueberry fruits.

## Supporting information

S1 Table Summary table of the transcriptome analysis at harvest, 21days post-harvest [dph] at 4°C and following 3 days shelf life at 18°C [24 dph] in ‘Bluecrop’ and ‘Legacy’. (DOCX)

S2 Table Summary data for Blastn alignments of the ‘Bluecrop’ unigene set queried against the tetraploid *V*. *corymbosum* ‘Draper’ [22] gene prediction set. (XLSX)

S3 Table Summary data for Blastn alignments of the ‘Bluecrop’ unigene set queried against the tetraploid *V*. *corymbosum* ‘Draper’ [22] gene prediction set. (ZIP)

S4 Table Data for the 765 differentially-expressed genes between harvest [H] and 24-days post-harvest [3_3] clustered into four model profiles based on the expression levels in ‘Bluecrop’ and ‘Legacy’ including Unigene ID, log fold-change, significance, and annotations. (ZIP)
