# Supplementary material for: De novo sequencing and analysis of the transcriptome of two highbush blueberry (Vaccinium corymbosum L.) cultivars ‘Bluecrop’ and ‘Legacy’ at harvest and following post-harvest storage
Source: PLoS One. 2021 Aug 2;16(8):e0255139. doi: 10.1371/journal.pone.0255139 (PMC8328333; doi:10.1371/journal.pone.0255139)
Supplement: S1 Table — (DOCX) [file pone.0255139.s001.docx]

S1 Table. Summary table of the transcriptome analysis at harvest, 21days post-harvest (dph) at 4ºC and following 3 days shelf life at 18ºC (24 dph) in ‘Bluecrop’ and ‘Legacy’.

|  | Before and after trimming and quality control | | | | | | | |
| --- | --- | --- | --- | --- | --- | --- | --- | --- |
|  | Bluecrop | | | | Legacy | | | |
| Sequences | Harvest | 21 dph | 24 dph | Leaf | Harvest | 21 dph | 24 dph | Leaf |
| Number of raw reads | 67717387 | 72118586 | 80410832 | 22557244 | 67910891 | 64930867 | 84403728 | 22772432 |
| Number of cleaned reads | 65955465 | 70162861 | 77642138 | 21868295 | 66826263 | 63855731 | 81854546 | 22411915 |
| Number of assembled transcripts | 273834 | 309212 | 316512 | 155656 | 293774 | 359820 | 347551 | 105387 |
